# 74. An unsolved mystery of multisystem disease

**DOI:** 10.1093/rap/rky034.037

**Published:** 2018-09-20

**Authors:** Claire Wood, Michael Reed, Meng May Chee

**Affiliations:** 1 Department of General Medicine, University Hospital Wishaw, Wishaw, UNITED KINGDOM; 2 Rheumatology Department, University Hospital Wishaw, Wishaw, UNITED KINGDOM

**Introduction:** Rheumatologists frequently manage patients with multisystem diseases. It is not uncommon for the diagnosis to be unclear initially, but in most cases the combination of clinical features, the course of the disease and targeted investigation confirms a diagnosis; allowing delivery of evidence-based treatments. However, there are some cases that do not fit a recognised disease pattern and in whom extensive investigation may not identify a unifying diagnosis. We describe the complex case of a 28 year old female who initially presented in 2011 with symptoms related to a pleural effusion and who has since developed an increasing number of manifestations of an as yet undiagnosed multi-system inflammatory disorder. Consequently, she has been seen by numerous specialties and had extensive investigation undertaken throughout the years. She has also had trials of different immune modulating treatments with little effect. She has remained a diagnostic challenge and involvement of tertiary centres has been necessary. This case highlights the unusual clinical course of this illness, the various investigations and trials of therapy, and the many cross-site and inter-specialty discussions we have had regarding this patient. We hope this case highlights a rare clinical entity that may allow a discussion at conference to improve care of this patient and other patients with similar presentations.

**Case description:** We present the case of LF, a 28 year old female with a past medical history of autism/Asperger’s syndrome, bilateral sensorineural deafness, migraines. She has one relative with rheumatoid arthritis (mother) but no other significant family history. She initially presented in November 2011 at 22 years old with breathlessness, pleuritic chest pain and a large right pleural effusion. Empyema was suspected and she was treated with antibiotics and a chest drain. Sampling revealed an exudative effusion and microbiology was negative. At the time of chest drain insertion the pleura was notably thickened. She was allowed home but re-presented in March 2012 with bilateral pleural effusions. An echo excluded heart failure, and she was again treated with antibiotics and discharged. Following these initial admissions she has had recurrent presentations with problems arising from different organ systems and has had involvement from rheumatology, respiratory, gastroenterology, dermatology, infectious diseases, ophthalmology, genetics and neurology. The timeline of her problems from 2011 to date is summarised as follows: November 2011: initial presentation with right pleural effusion March 2012: bilateral pleural effusions April 2012: anterior uveitis, treated with prednisolone drops. Developed arthralgia in knees, shoulder and elbows. Throughout 2012: frequent presentations to hospital with recurrent symptomatic pleural effusions leading to medical thoracoscopy and biopsy December 2012: initial referral to rheumatology: described Raynaud's phenomenon and arthralgia (RF, anti-CCP, ANA, ANCA and HLA-B27 all negative. Normal complement). Treated with combination of steroids and hydroxychloroquine. March 2013: admission with severe pleurisy and worsening effusions managed with higher dose steroids. June 2014: widespread eczema-like rash which resolved before dermatology review. August 2014: ongoing rheumatology review, referred to National Lupus Centre in London for further opinion. Possibility of an auto-inflammatory disorder raised October 2014: seen by gastroenterology with intermittent abdominal pain and suggestionof colitis on CT. Faecal calprotectin, colonoscopy and biopsies suggestive of early inflammatory bowel disease so started on pentasa and budesonide. October 2014: discussed with specialist in auto-inflammatory disorders and history felt atypical of a hereditary periodic fever or auto-inflammatory syndrome. Bloods sent for genetic sequencing which did not show evidence of any known mutations associated with auto-inflammatory conditions. March 2015-February 2016: seen by neurology with peripheral neuropathic symptoms initially thought secondary to nutritional deficiencies. September 2015: further episode of anterior uveitis. September 2015: seen by clinical genetics. Array CHG shows deletion of chromosome 15 and duplication of NLGN4 gene on X chromosome implicated in causing autism and Asperger’s but not thought to account for her other medical presentations. January 2016: admission with marked ascites and extensive nodular peritoneal enhancement on CT. Diagnostic laparotomy and biopsy undertaken in February 2016 and reviewed by infectious diseases who felt TB was unlikely. February 2016: develops increasing problems with swallow and barium study shows oesophageal stricture but further OGD shows severe oesophagitis only. 2016-2017: frequent admissions with recurrent ascites requiring drainage. October 2016: given empirical treatment for TB as peritoneal and pleural biopsies had shown granulomatous disease (although AAFB repeatedly negative). April 2017: admission with septic shock and an incarcerated hernia requiring ITU. November 2017: case referred to tertiary centre in Glasgow. February 2018: admission with progressive breathlessness, deteriorating PFTs and CT appearances. Respiratory advice to refer for consideration of lung transplant. March/April 2018: repeat upper and lower GI endoscopy showed ileitis but no granuloma and the question of Crohn’s disease was raised again. May 2018: seen in tertiary centre and case discussed with auto-inflammatory disease and interferonopathy specialists in Edinburgh and USA She has been extensively investigated through the years. The following summarises her testing: CRP usually 20-40 mg/L and ESR 25 mm/hr, Urine for 5HIAA and catecholamines negative, ferritin 33 ng/ml microbiology: Pleural and ascitic fluid repeatedly negative on microbiology culture, sputum, faeces, pleural fluid, ascitic fluid, broncho-alveolar lavage, pleural biopsy and peritoneal biopsy all negative on testing for AAFB, HIV, Hepatitis B and Hepatitis C negative, syphilis antibody negative, filarial ELISA, brucella IgG and IgM, leishmania, schistosomal ELISA, strongyloides ELISA, toxoplasma all negative. Pro-calcitonin 0.05 ng/ml. Immunology: repeated auto-antibody testing - negative RF, anti-CCP, ANCA, C3, C4. ANA 1:160, ENA negative, serum ACE normal, C1 inhibitor normal, lymphocyte subsets generally reduced, normal levels of CH100 and AP100 (classical and alternative complement pathways), IgG, IgA and IgM raised. Genetic testing: HLA B27 negative, Array CHG shows deletion of chromosome 15 and duplication of NLGN4 gene on X chromosome. Cardiorespiratory: Thoracoscopy showing inflammatory lesions on the diaphragm with thickened and inflamed pleura. Pleural biopsy showed severe inflammation with lympho-plasmacytic infiltrate and reactive surface mesothelium. PFTs showing progressive restrictive defect (FVC 26% predicted, FEV1 24% predicted). Repeated chest CT showing progressive bilateral consolidative foci and pleural effusions. Organising pneumonia demonstrated recently. Bronchoscopy with BAL negative for microbiology and AAFB testing, six minute walk test showing desaturation on walking, repeated echocardiograms showing normal LV and RV function. GI: barium swallow showed oesophageal stricture, repeated upper GI endoscopies showing hiatus hernia and oesophagitis, repeated colonoscopy initially showing apthous ulcers and later ileitis, faecal calprotectin positive (480 ug/g at highest), MRI not tolerated but CT small bowel showed gross ascites and extensive nodular peritoneal enhancement. Diagnostic laparotomy showing pus like ascitic fluid negative on microbiology and AAFB culture. Other Imaging: DEXA showed osteoporosis, MRI head and inner ear to look for inflammation of inner ear structures negative. Hand x-rays, MRI both hips and L knee showed no features of inflammatory arthritis. Pathology: peritoneal biopsy showed a granulomatous inflammatory infiltrate containing granulomas with central necrosis but mycobacteria stains were negative, pleural biopsy showed severe inflammation with lympho-plasmacytic infiltrate and reactive surface mesothelium. Staining of rectal biopsy for amyloid negative and no excessive staining of IgG or IgG4. Over the years she has had trials with a number of different treatments as described below: pused on and off with a variable response. At one stage she became significantly cushingoid with this and recent DEXA has shown osteoporosis hydroxychloroquine used with some response from September 2012 but stopped during admission in March 2013 with a worsening pleural effusion. Switched to azathioprine as a steroid sparing agent in May 2013 but not tolerated. Methotrexate started June 2013 again for steroid sparing but ineffective so discontinued. August 2014: brief trial of colchicine. October-December 2014 stopped due to diarrhea. Empirical TB treatment with rifater (rifampacin, isoniazid, pyrazinamide), ethambutol and pyridoxine in September 2016 for nine months. Current thinking is that she has an auto-inflammatory condition or an interferonopathy, although the presentation is atypical for the known single gene mutation auto-inflammatory diseases. The current plan for her management is a trial of anti-IL1 therapy, and in the event of failure of this, a JAK inhibitor.

**Discussion:** This case highlights the challenges associated with managing rare diseases. The multisystem nature and the fluctuating course of this patients's illness led to her being seen by multiple different medical specialties and each had a different approach to further investigation or management. To our knowledge, this is a unique case in terms of the manifestations of the systemic disorder and the lack of identified mutation seen in recognised auto-inflammatory conditions. In managing this complex case, whilst attempting a series of treatments, our approach has also involved attempts to coordinate efforts with colleagues in other specialties and to seek assistance from national experts to see if we can come up with a more satisfactory management plan. Our patient has displayed a variable response to steroid therapy. She has had multiple courses of antimicrobials, despite no definitive evidence of infection. She has also had no response to methotrexate, colchicine or hydroxyxhloroquine. Her lung disease has progressed to the extent that she may be considered for lung transplant. There remains uncertainty in considering the best way forward in terms of pharmacological management. Anti-IL1 therapies have a role in treatment of auto-inflammatory conditions, with reports of success. Many respond to steroid therapy, some respond to colchicine, and some respond to DMARD therapies such as methotrexate. The inability of intial genetic testing to identify a known associated mutation has hindered diagnosis and management. However, the number of recognised mutations is increasing, and it is recognised that a large proportion of cases of auto-inflammatory conditions will not possess a known mutation. Our intention is to embark on IL-1 therapy after awaiting further genetic testing. Another option may be a JAK inhibitor.

**Key Learning Points:** Patients with rare multi-system inflammatory disorders are challenging to manageIt is important to investigate these patients in a targeted manner to try to narrow the differential diagnosisIf condsidering an auto-inflammatory condition, consider genetic analysis, however this may not be conclusiveInter-specialty collaboration is vital. Tertiary referral and/or expert opinion should be sought.

**Disclosure: C. Wood:** None. **M. Reed:** None. **M. Chee:** None.

